# Forced Duction Testing for Management of Small Orbital Floor Blowout Fractures

**DOI:** 10.7759/cureus.93001

**Published:** 2025-09-23

**Authors:** Jason V Djafar, Lloyd R Kopecny, Isobel Yeap, Alex Pitman, Ian C Francis

**Affiliations:** 1 Department of Ophthalmology, Faculty of Medicine, University of New South Wales, Sydney, AUS; 2 Department of Plastic and Reconstructive Surgery, Northern Beaches Hospital, Sydney, AUS; 3 Department of Radiology, Northern Beaches Hospital, Sydney, AUS; 4 Department of Ophthalmology, Prince of Wales Hospital, Sydney, AUS

**Keywords:** forced duction testing, minimally invasive management, orbital floor blowout fracture, orbital fracture, restricted upgaze

## Abstract

Forced duction testing (FDT) is a diagnostic clinical test of extraocular muscle function employed to confirm the mechanical and restrictive nature of a patient’s defective upgaze. Orbital floor blowout fractures (OFBFs) may lead to bony entrapment of the inferior orbital muscles and connective tissue, resulting in restricted vertical gaze and early or late enophthalmos. Management of OFBFs aims to restore normal ocular anatomy and physiology and may require definitive surgical intervention if there is significant entrapment and large fractures. The authors report a 19-year-old man with a small right OFBF due to a rugby football injury, who presented with painful restricted upgaze. High-resolution orbital computed tomography (CT) demonstrated minor impingement of the inferior rectus muscle by the fractured thin orbital floor bone. FDT was performed in the Emergency Department in an attempt to confirm the restricted vertical movement of the involved eye and then to release the impinged inferior rectus muscle. This was achieved immediately and successfully, with rapid resolution of the patient’s painful restricted upgaze. Hence, in selected cases of small OFBFs, FDT may have both a diagnostic and effective therapeutic role.

## Introduction

Orbital floor fractures were first described in 1844 [1]. The term “orbital blow-out” fracture was described by Smith and Regan in 1957 [2], referring to an orbital floor fracture caused by a sudden increase in intraorbital pressure. Initial imaging of orbital floor blowout fractures (OFBFs) was carried out using plain orbital X-rays, which were superseded with the advent of computed tomography (CT) scans [3].

The entrapment of the inferior rectus or inferior oblique muscles with their surrounding orbital connective tissues is a concerning possible complication of OFBFs. This may present as vertical diplopia, painful restriction of vertical gaze, and rarely the oculocardiac reflex. Forced duction testing (FDT) is a test of extraocular muscle restriction performed by first applying local anaesthetic to the conjunctiva and then mobilising the globe using forceps attached to the insertion of the relevant muscle [4]. In OFBF with entrapment, the examiner observes an inability to move the patient’s eye freely, reflective of the underlying globe tethering and restricted movement in this pathology. In the paediatric population, bony elasticity may cause the fractured orbital floor to snap back into position, simulating a “trapdoor” that incarcerates these muscles [5,6]. To avoid a Volkmann-like ischaemic contracture, urgent surgery is indicated [7].

Many OFBFs are not associated with entrapment and may be observed with follow-up in 1-2 weeks until orbital oedema, haemorrhage, and motor nerve palsy subside, enabling subsequent assessment of extraocular movements and enophthalmos. Indications for delayed surgery include enophthalmos greater than 2-3 mm, an orbital floor defect on CT greater than 2 cm², or one that involves 50% or more of the orbital floor surface [8]. Patients who do not meet surgical criteria after a period of observation have similar outcomes when managed conservatively [9,10].

Ophthalmological assessment is always indicated to exclude concomitant ocular trauma, which is reported in up to 75% of cases [11,12]. Richani et al. (2019) presented a series of 512 patients with orbital floor fractures, whereby 14% had severe ocular trauma and vision loss [13]. Early operative repair is contraindicated when hyphaema, retinal tears, globe perforation, or contralateral blindness is present.

## Case presentation

A 19-year-old man presented to the emergency department (ED) of a tertiary referral teaching hospital in Sydney, Australia, following a right orbital injury whilst playing rugby that same day.

**Figure 1:**
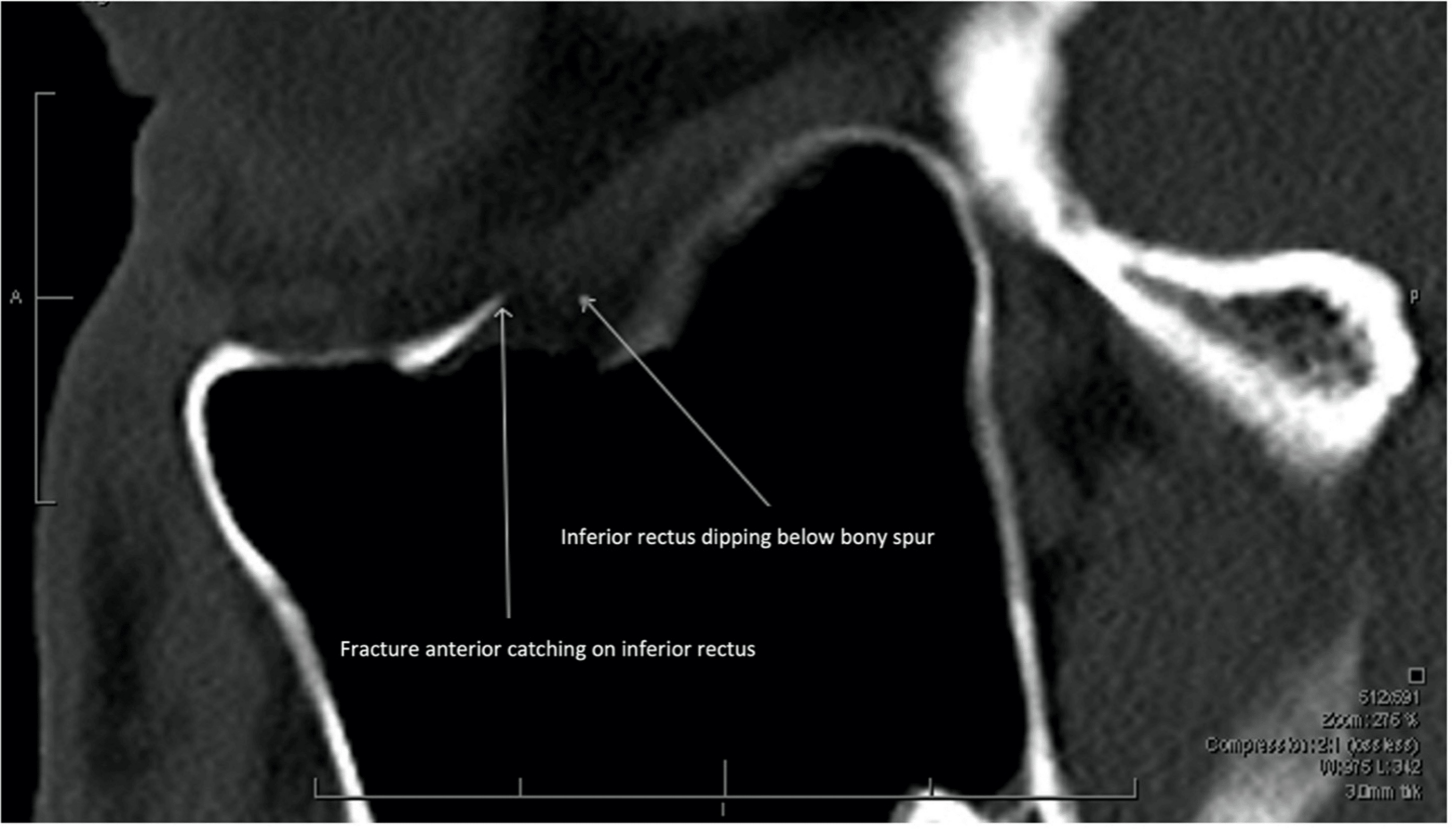
Parasagittal CT Orbits (Bony Windows), Prior to Forced Duction Testing. Note the anterior lip of the fractured bone impinging on the anteroinferior aspect of the inferior rectus, with modest orbital fat herniation.

Vertical diplopia was present, related to defective elevation (-2/4) and depression (-1/4). The patient had severe ocular pain with upgaze of the right eye. Periorbital ecchymosis was evident. Using the Luedde proptometer [11], 3mm of right proptosis was observed (18mm right, 15mm left). Light touch sensation in the distribution of the ipsilateral infraorbital nerve was reduced. The ophthalmological examination was otherwise normal. Orbital CT demonstrated a small OFBF measuring 1.44 cm² in the mid-orbital floor. The anterior lip of the fractured bone impinged on the anteroinferior aspect of the inferior rectus muscle, with herniation of orbital fat (Figure 1).

**Figure 2:**
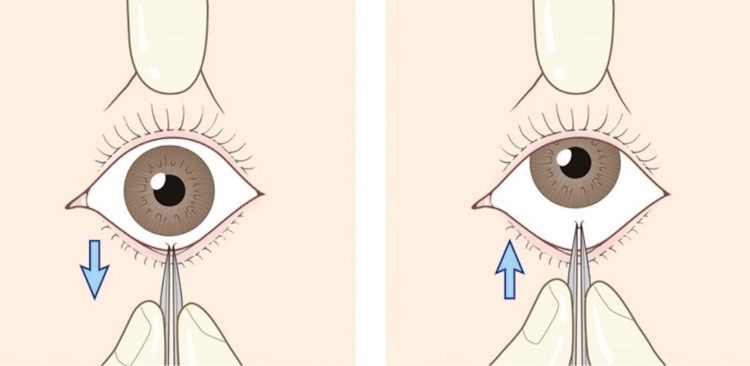
Diagram of Forced Duction Testing to Examine Extraocular Muscle Entrapment. The examiner uses forceps to grasp the anaesthetised conjunctiva overlying the attachment of the inferior rectus muscle. The examiner assesses the patient’s defective upgaze and gently attempts to release the entrapped inferior rectus muscle. (reproduced from Kim & Jeong, 2016 (6), used under the Creative Commons Non-Commercial Licence, CC BY 3.0)

The corresponding author (ICF) had previously managed a similar case, where the patient’s vertical diplopia and defective eye elevation resolved with FDT. This was performed on a 28-year-old man who had sustained a punch to the orbit, resulting in radiological and clinical evidence of an OFBF. At that time, CT was not available, and a plain orbital X-ray was employed to confirm the clinical diagnosis radiologically. Using a standard diagnostic FDT, the patient’s OFBF resolved immediately and uneventfully (Figure 2) [6]. 

Hence, with informed consent, for the current patient, FDT was performed in the ED. The indications in general for this procedure include a small OFBF (< 2 cm²), radiological evidence of bony impingement by orbital floor bone, and associated defective elevation of the patient’s affected eye. The procedure should be performed in the ED, where ECG monitoring is readily available to recognise the possibility of the oculocardiac reflex. 

The authors’ protocol for Forced Duction Testing to manage small Orbital Floor Blowout Fractures (FDT-OFBF) is outlined below (Table 1).

**Table 1:** Protocol for Forced Duction Testing for Management of Small Orbital Floor Blowout Fractures.

Protocol
Informed consent
Electrocardiograph (ECG) monitoring
Instillation of oxybuprocaine topical anaesthetic eyedrops
Placement of lid speculum
Infiltration of 2% lignocaine transconjunctivally into the insertion of the inferior rectus, using a 30-gauge needle
Application of toothed Adson forceps to inferior rectus insertion
Performance of standard Forced Duction Testing, assessing the patient’s defective elevation of the eye, and releasing the entrapped inferior rectus

Following FDT, there was immediate resolution of the patient’s diplopia. The patient’s defective elevation of that eye returned to near-normal and was pain-free. The patient remained comfortable throughout the procedure, without evidence of arrhythmias or haemodynamic instability. In fact, he broke out into a smile on an attempted, successful upgaze. The patient was admitted overnight to monitor for and exclude retrobulbar haemorrhage, employing standard neurological observations for pain, visual acuity, pupil reactivity, and proptosis.

**Figure 3:**
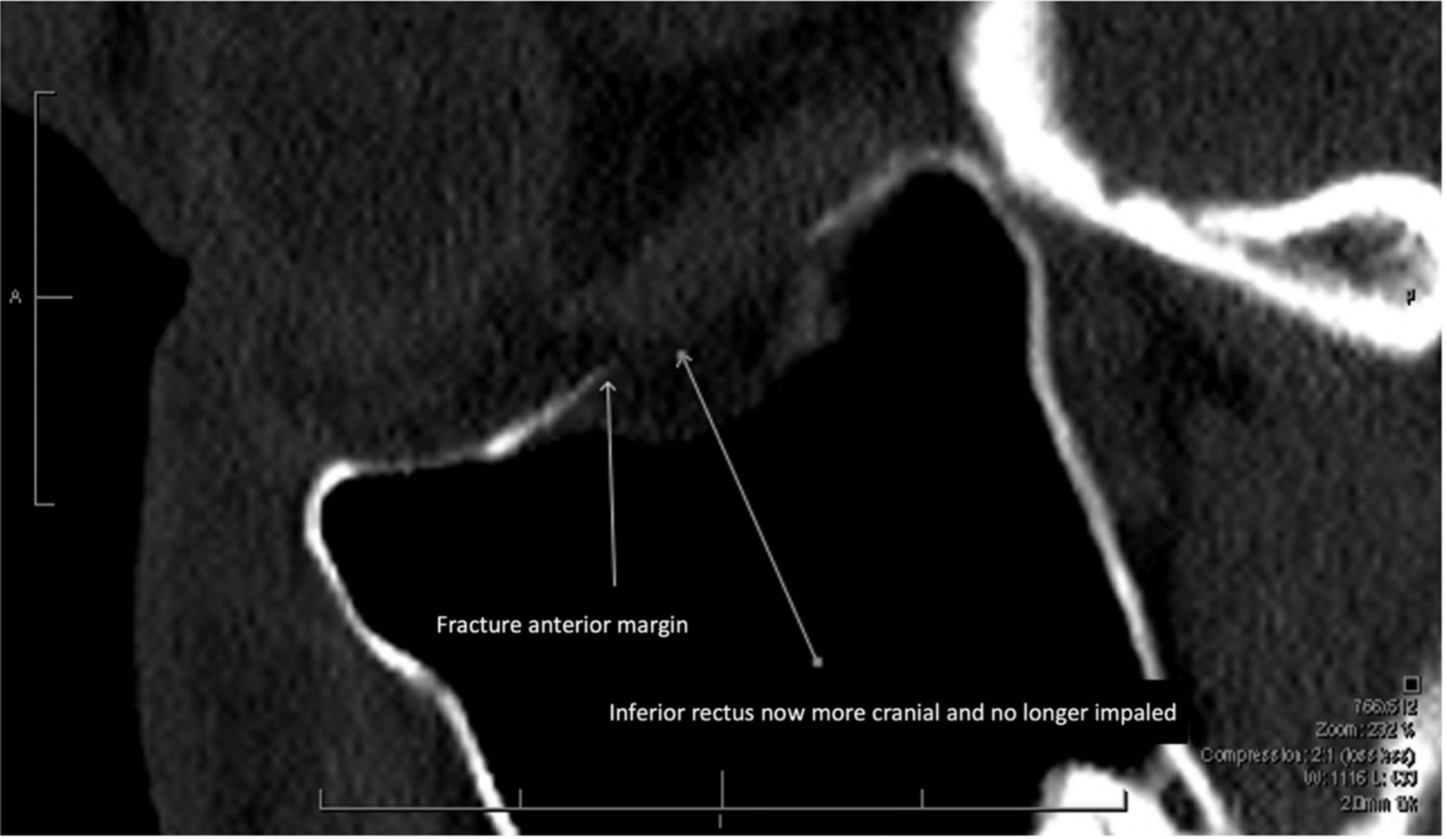
Sagittal CT Orbits (Bony Windows), Following Forced Duction Testing for Resolution of Inferior Rectus Impingement. The inferior rectus is now more cranial and no longer impaled by the anterior margin of the fractured bone.

By the next morning, his defective right ocular elevation had improved (-1/4), and depression had returned to normal (0/4). Repeat CT confirmed satisfactory OFBF reduction (Figure 3). After being provided with instructions for intensive eye movement exercises, the patient was discharged for outpatient follow-up.

Seven weeks following therapeutic FDT, the patient reported no diplopia. Defective right elevation was reduced to less than -0.5/4. Infraorbital nerve sensation had returned to near normal. The patient’s proptosis had normalised as measured by Luedde proptometry (14 mm right, 15 mm left).

## Discussion

This report demonstrates that FDT for OFBFs (FDT-OFBF), performed under topical and local infiltrative anaesthesia, may be used to resolve inferior orbital muscle impingement by the bones of the orbital floor. This simple, inexpensive, and minimally invasive technique may be performed in the ED where cardiac monitoring is readily available. It can represent an initial step in the conservative management of OFBFs, as opposed to immediate triaging for standard operative OFBF repair in the operating room (OR).

Suitable candidates for FDT-OFBF should include patients with small OFBFs who have radiological evidence of bony impingement of the inferior orbital muscles and connective tissue, with associated restriction in vertical eye movements. Small OFBFs have previously been described in the literature as those with an estimated fracture area between 1 and 2 cm² [14]. The authors have suggested the various advantages and disadvantages of FDT to manage extraocular muscle impingement in Table 2. FDT-OFBF avoids almost all the risks and complications of definitive surgery on OFBFs in the OR.

**Table 2:** Suggested Advantages and Disadvantages of Forced Duction Testing to Manage Small Orbital Floor Blowout Fractures. ^a^ Diplopia, postoperative hyperaesthesia, ectropion, enophthalmos, are commonly reported in the literature. Vision loss, often due to infraorbital haemorrhage, is rare but serious and may require repeat surgery. Other complications include those related to a prolonged hospital stay (urinary tract infection, pneumonia, hypertensive crisis) [15,17].

Advantages of Forced Duction Testing	Disadvantages of Forced Duction Testing
Definitive and immediate re-establishment of normal eye movements	General anaesthesia is required in children.
Performance in an Outpatient/Emergency Department setting under local anaesthesia	Potential trauma by forced duction testing to the inferior rectus muscle insertion
Avoidance of orbital floor implants for reconstruction (alloplasts, autografts)[15]	Theoretical risk of triggering the oculocardiac reflex with extraocular muscle traction (although no case reports to date in the literature)[16]
Avoidance of common post-operative complications related to open intervention^a^	
Significant time and cost savings	
Operative surgery in operating theatre remains viable if the initial trial of forced duction testing for orbital floor blowout fractures fails	

As plastic surgeons, ocular plastic surgeons, and maxillofacial surgeons are regularly faced with the management of OFBFs, diagnostic FDT may be an effective therapeutic option to treat selected OFBFs. Following FDT, rapid resolution of pain, diplopia, and defective ocular elevation and depression meant this patient avoided invasive surgery under general anaesthesia. If forced duction testing fails to provide symptom resolution, definitive surgery in the OR is still available.

The oculocardiac reflex (OCR) is reported to occur in 14-90% of patients undergoing strabismus surgery [16], attributed to the relatively long duration of extraocular muscle traction that occurs in strabismus surgery. Indeed, there are no reports of the OCR related to FDT, despite FDT being a relatively commonly performed procedure. By comparison, the almost instantaneous duration of FDT-OFBF renders the likelihood of the OCR almost negligible.

Trauma to the inferior rectus and inferior oblique muscles with therapeutic FDT is unlikely, since the mechanical force applied to the anaesthetised inferior rectus insertion is similar to that applied when carrying out diagnostic FDT. Indeed, there are no literature reports documenting such trauma. Finally, if impingement does not resolve with FDT-OFBF in the outpatient setting, definitive OFBF surgery in the OR is indicated and may be undertaken uneventfully.

## Conclusions

In summary, FDT-OFBF offers an alternative, simple, and non-invasive management strategy for selected patients with OFBFs. FDT-OFBF may be considered as a possible and effective addition to the surgical repertoire of plastic surgeons, ocular plastic surgeons, and maxillofacial surgeons for the management of select OFBFs. When successful, FDT avoids definitive surgical management of the OFBF along with its potential associated complications. Further research, such as a prospective multicentre trial, should refine the indications for, the outcomes of, and the complications associated with FDT-OFBF.
